# The process of co‐design for a new anxiety intervention for autistic children

**DOI:** 10.1002/jcv2.12255

**Published:** 2024-08-13

**Authors:** Tasha Cullingham, Una Rennard, Cathy Creswell, Damian Milton, Karen Leneh Buckle, Lucie Godber, Kate Gordon, Michael Larkin, Jonathan Green

**Affiliations:** ^1^ Manchester University NHS Foundation Trust Manchester UK; ^2^ Expert by Experience; ^3^ Departments of Experimental Psychology and Psychiatry University of Oxford Oxford UK; ^4^ Tizard Centre University of Kent Canterbury UK; ^5^ Division of Psychology Communication and Human Neuroscience Faculty of Biology Medicine and Health University of Manchester Manchester UK; ^6^ Department of Neuropsychology Berkshire Healthcare NHS Foundation Trust Bracknell UK; ^7^ College of Health and Life Sciences Aston University Birmingham UK; ^8^ Division of Psychology and Mental Health Faculty of Biology Medicine and Health University of Manchester Manchester UK

**Keywords:** autism, CBT, child anxiety, co‐design, co‐production, neurodiversity, parent‐led intervention

## Abstract

**Background:**

Mental health difficulties are common for autistic people; however, almost no interventions have been co‐designed with the autistic community. Co‐design has the potential to add important insights from lived experience into intervention design, but there are currently limited examples of how rigorously to undertake this practice. This paper details a worked model of co‐design and its process, focussed on adapting an evidenced parent‐led intervention for non‐autistic child anxiety (HYC), to meet the needs of young autistic children. The aim is to provide an example of co‐design, integrating autistic, parental, academic, clinical, experience and expertise.

**Methods:**

Using prior literature and theory, including Experience‐Based Co‐Design, we developed an iterative and collaborative process between the research team and an expert reference group (ERG). The research team comprised autistic and non‐autistic members. The ERG included parents (autistic and non‐autistic) of autistic children with anxiety problems, autistic adults with experience of anxiety problems, and clinicians with experience supporting autistic children with anxiety problems. The ERG and research team reviewed information from qualitative research interviews with autistic children with anxiety problems and their parents along with information from clinical experience and the academic literature to reach consensus on the adapted intervention design.

**Results:**

The creation of a truly co‐designed intervention that includes a neurodiversity‐affirmative perspective, alongside CBT techniques. With anxiety problems experienced by autistic children being framed by combining the impacts of being neurodivergent in a neurotypical world, developmental science and well known cognitive behavioural models of child‐anxiety.

**Conclusion:**

Co‐design can help to integrate multiple perspectives and result in the creation of interventions that are potentially relevant and acceptable to autistic people, their family members, and clinicians.

## Graphical abstract

Co‐design of new interventions, including autistic interventions, is now strongly advocated; but there are very few reports to date of the actual process of such co‐design in practice with the autistic community. We report on such a co‐design process in practice, following current guidance, for an anxiety intervention for young autistic children. We show how co‐design process can help to integrate multiple perspectives and result in the creation of novel co‐created interventions.

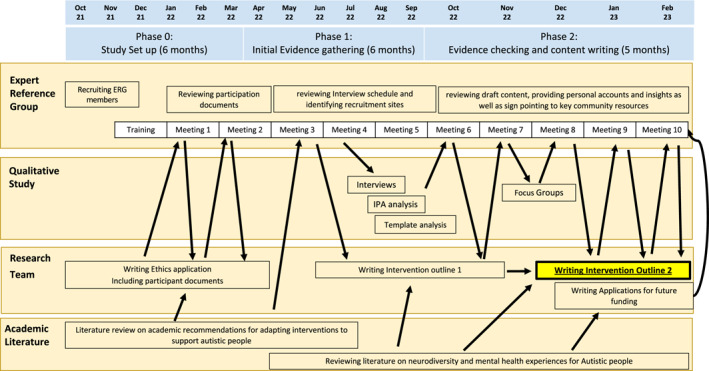

**Key points**

**What's known?**

- Co‐production is often recommended, yet to date there are limited methodological papers that explore how to successfully co‐produce mental health interventions with autistic people. We aimed to exemplify some methods in practice and to report on successes, value, and challenges.

**What's new?**

- We undertook a systematic and Experience‐Based Co‐Design (EBCD) methodology within a sensitive area of child mental health care; in adapting an existing therapist enabled parent‐led intervention for anxiety in non‐autistic children to be suitable for autistic children. We detailed theory, co‐design process and resulting successes and challenges.

**What's relevant?**

- The co‐design process was successful in its aim and was considered to have added essential value. It allowed an integration of autistic perspectives on anxiety and its development, theory from autistic scholarship, with developmental science and clinical experience on anxiety within autistic development. It achieved a consensus outcome on the adaptation and increased mutual engagement and understanding between stakeholders.

## INTRODUCTION

In the last 2 decades, what has been called a ‘participatory zeitgeist’ (Palmer et al., 2019) has accelerated generally across public policy (Brandsen & Honingh, 2018) and health care (Donetto et al., 2015). This has been embodied for healthcare in Berwick's call (Berwick, 2016) for a move beyond what he characterises as ‘Era 1, professionally determined’ and ‘Era 2, technocratically measured’ approach, into an ‘Era 3, moral’ healthcare in which ‘…(T)he more patients and families become empowered, shaping their care, the better that care becomes….’ How this is enacted in practice is often discussed in terms of co‐design of interventions and services (Brandsen & Honingh, 2018; Palmer et al., 2019), at its core a collaborative partnership between key stakeholders often including service users, the public, clinicians, and researchers. Its features include ongoing systematic processes from the foundation of a project to integrate diverse perspectives towards a shared outcome, thus contributing to more acceptable, relevant, and effective research outputs. To be meaningful and not tokenistic, however, such an approach needs to be embodied in detailed practical processes and grounded in iterative action orientated research and practice (Hewitt et al., 2023; Moll et al., 2020).

Within this general movement, autism research and practice has arguably been relatively slow in developing participatory work with service users and the autism community, including parents of autistic children (Fletcher‐Watson et al., 2019). However, recent years have seen increasing impetus towards this, stimulated by advocacy from the neurodiversity movement and recognition of the need for this from many in the clinical and research communities (Bertilsdotter Rosqvist et al., 2019; Chown et al., 2017; Leadbitter et al., 2021; Milton & Bracher, 2013.). Fletcher‐Watson et al. (2021) set out ethical as well as practical arguments for co‐design with autistic people and for future research to involve autistic voices. Alongside this, specific guidance on best practice for co‐production involving autistic and non‐autistic people has evolved (Fletcher‐Watson et al., 2019; Nicolaidis et al., 2019; Stark et al., 2021).

This paper describes the detail in practice of such a co‐design process, used in the development of a new anxiety intervention for young autistic children. We have utilised the guidance and recommendations established by autistic co‐production groups as above, and we report on its processes, successes and challenges, using process and output documentation and participant reflection.

### The intervention focus: Intervention for anxiety in young autistic children

Anxiety problems are the most commonly occurring mental health difficulty for autistic people (Vasa et al., 2020). They have an early onset (Solmi et al., 2022) and can have life‐long implications and negatively impact both the child and their family. In England, National Institute for Health and Care Excellence, 2013 (NICE) guidance [CG170] (National Institute for Health and Care Excellence, 2013) recommends that co‐occurring mental health conditions experienced by autistic people, such as anxiety problems, should be managed by adapting existing interventions evidenced in the non‐autistic population. Whilst adaptations of this kind have been made, (e.g., Wood et al., 2020), true co‐production of such an adaptation has not to our knowledge been reported. Moreover, since the developmental origins, phenomenology and clinical presentation of anxiety in autistic children is substantially different to that in neurotypical children (Shephard et al., 2019), co‐design is a way optimally to refine and focus intervention processes and targets for this group.

### Choice of a therapy model for adaptation

In the development of this project, parents of young autistic children often reported that they felt left alone to help their child and wanted to know how best they could help, indicating a need for parent‐focused approaches. Other intervention science in autism has shown that a parent‐mediated approach is not only highly acceptable in meeting family needs (Leadbitter et al., 2020), but also has the best evidence for sustained long‐term developmental benefits for the child (Pickles et al., 2016), benefits themselves mediated by intervention changes within the parent‐child dyad (Carruthers et al., 2023). This evidence, and NICE guidance [CG170] (National Institute for Health and Care Excellence, 2013), informed our selection of the ‘Help Your Child’ (HYC) programme as the focus for adaptation for an anxiety intervention in young autistic children. HYC is parent‐led, it has been shown clinically effective and cost‐effective in reducing anxiety in pre‐adolescent non‐autistic children (Creswell et al., 2017; Thirlwall et al., 2013), and been successfully implemented in England's children's mental health services (Wood, 2021). HYC had recently been transposed to an online version via a co‐design process with parents (Hill et al., 2022), with results suggesting that the online approach achieved at least as good outcomes in non‐inferiority tests, and had added potential to further increase access to treatment through increased efficiency (Hill et al., 2022). We therefore selected for adaptation the resulting ***Online*** ***Support*** ***and Intervention for child anxiety (OSI)***. OSI is an 8‐session interactive web‐based intervention (including one welcome module and one follow‐up module). The content supports parents to help their children overcome problems with anxiety through simple interactive and multi‐modal content based on cognitive behavioural principles (Hill et al., 2022). Parents have weekly brief phone/online calls with a therapist to individualise the content (see Hill et al., 2022).

## METHODS

***Co‐Design*** To adapt OSI to meet the needs of autistic children and their families, we combined multi‐perspective qualitative data with processes and strategies adapted from experience‐based co‐design (EBCD, Donetto et al., 2015), health‐care co‐design (Hewitt et al., 2023; Moll et al., 2020) and autism‐specific models for participatory research (Fletcher‐Watson et al., 2021). Experience‐Based Co‐Design is described as a ‘participatory research approach that draws upon design tools and ways of thinking in order to bring healthcare staff and patients together to improve the quality of care’. We constructed a process whereby insights from key stakeholder groups could be integrated; in this case parents, children, autistic adults, academics, and clinicians. Using these insights, we aimed to adapt OSI and create method and content for an online, brief, parent‐led anxiety intervention for autistic children (aged 5–12). Our approach to some key principles for autistic co‐design are detailed in (Table S1).

***Embedded Qualitative Study*** As part of the co‐design, an embedded qualitative study used Interpretive Phenomenological Analysis and Template Analysis to draw out key themes in children and parents' experiences on the understanding of anxiety problems among autistic children. Semi‐structured interviews were undertaken with 10 parents of autistic children with anxiety problems (children aged 8–12 years, median age 10) and 9 autistic children with anxiety problems (children aged 8–12 years, median age 10). This Qualitive Study will be fully reported separately. For this current report, we focus on how this qualitative method was used and integrated within the co‐design process. Further details on the qualitative study are included in the Supporting Information (SI, pages 1‐5). Later in the co‐design process, additional parent focus groups were conducted to review the initial draft adaptation.

### Collaborating teams

***The Research Team*** The research team included a non‐autistic Patient and Public Involvement (PPI) expert who is a parent of an autistic young man with longstanding anxiety problems with extensive experience of involvement with national NIHR PPI initiatives (UR); an autistic researcher who has research expertise in autism, particularly critical autism studies, who is also a parent of an autistic adult with a learning disability (DM); a non‐autistic consultant clinical psychologist and expert in child anxiety interventions (CC); a non‐autistic expert in qualitative methods and co‐production methodology (ML); a non‐autistic child and adolescent psychiatrist with expertise in autism science, intervention development and clinical practice (JG); and a non‐autistic neurodivergent (dyslexic) clinical psychologist with clinical expertise in supporting autistic children with mental health difficulties and their parents (TC).

***The Expert Reference Group (ERG)*** At the centre of the co‐adaptation process was an ERG consisting of four parents (autistic and non‐autistic) of autistic children with anxiety problems, an autistic young person (<18 years old) with experience of anxiety problems, and two non‐autistic clinicians with experience supporting autistic children with mental health difficulties. Five members of the ERG (LG, KLB, DM, UR, KG) are co‐authors on this paper. Two members of the Research team, PPI lead (UR) and lead researcher (TC), co‐chaired the ERG. An expert in critical autism studies from the Research team (DM) was also included as a member. For ERG membership the research team sent out invitations for expressions of interest, along with criteria, through local and national parent carer forums and autism charities, including the research charity Autistica. The research team agreed a priori selection criteria which included balancing experience, ensuring we had a parent of a child who was currently experiencing anxiety problems, autistic and not‐autistic parents, autistic adults with and without experience of mental health difficulties, as well as an autistic young person who had experienced anxiety problems. Interested applicants were invited to have a conversation with a member of the research team to discuss the role and answer any questions. Selection was guided by the agreed criteria. Prior to the first meeting, ERG members were provided training and support, which is detailed in sections below.

***Co‐constructed ERG structure*** The ERG meeting structure was flexible, and created by the ERG rather than being pre‐conceived by the research team, an example of joint ownership of key decisions (Hickey et al., 2018). For example, the original research plan suggested quarterly ½ day meetings; however, the ERG decided that regular shorter monthly meetings would be more effective. The general meeting structure was established within training sessions and initial meetings. All sessions had a pre‐sent informal agenda, regular breaks and check‐ins. The meetings would open and close with reflections, content was shared via PowerPoint, with time for discussion and critique. Processing breaks were given after intensive content was delivered.

***Facilitation of equitable communication and power sharing*** The ERG co‐chairs met regularly throughout the project to reflect on their own chairing and how they were contributing to, or dismantling, power imbalances; so as to minimise the impacts of power differentials (Farr, 2018). Through this reflection process the co‐chairs decided to take on different roles within the ERG (for example, to facilitate open and honest critique, the co‐chair with clinical and academic experience would present the content and the co‐chair with lived experience would lead the discussion). Both co‐chairs provided open reflections on their worries and hopes about chairing such a group, as well as on‐going challenges and successes of the project during the ERG meetings' open and closing reflections. The aim was to place equal expectations around honesty and sharing for all members of the ERG. Within the ERG, efforts were made to create balanced communication, giving weight to all perspectives; this was again regularly reviewed by the co‐chairs in their reflection meetings. This included flexible modes of communication, participation, and support, including different ways to input such as via group conversations, e‐mails, one‐to‐one phone calls or via drop in sessions. Within the group meetings options around communication were always provided, such as the option to have cameras on or off, the use of online meeting features such as hands‐up, and with different communication styles (for instance, verbal or written in the chat function) considered equally. At all meetings, an invitation to be critical was reiterated, and differences of opinion were openly discussed and welcomed. Minutes were taken at every ERG meeting and key points summarised; the ERG members then checked these for accuracy. The minutes and summaries were shared with the research team after a 48‐h review period.

### Co‐design process

**FIGURE 1:**
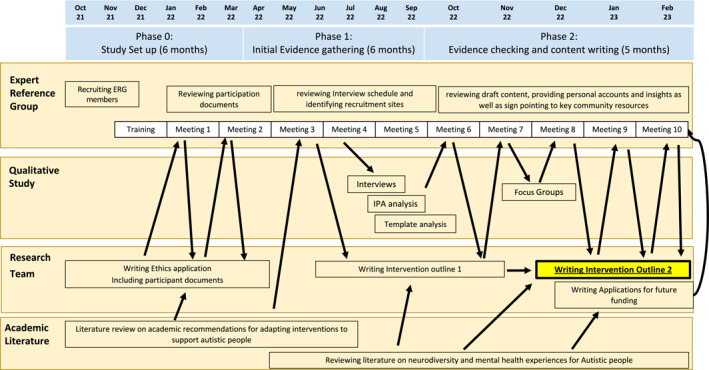
Iterative co‐design process for intervention development.

Central to the co‐design was an iterative feedback process between research team and ERG (Figure 1).

Information was integrated from multiple sources, including: the background research and theory literature; research team academic and clinical experience; ERG experience; data from the embedded qualitative study. In iterative fashion, and using deliberative practice (Donetto et al., 2015), the Research team developed an evolving ‘key insights’ documents, capturing learning from all sources, including the Qualitative Study (see SI page 1–5), and highlighting discrepancies. TC led on these documents to develop draft content which was reviewed by the ERG and the Research Team. Any component of the proposed adapted intervention that the ERG felt had the potential to be problematic was explored and discussed in detail. Following detailed discussion and problem‐solving, if the ERG still expressed concerns this was recorded as not to be included within the adapted intervention (an example of this was the exclusion of NICE‐recommended emotion‐recognition training, see Table S2).

If there was a disagreement between stakeholders the ultimate decision‐making responsibility would fall on TC and the research team, prioritising insights from those with lived experience. However, in practice we found this was not needed as the ERG and the research team found no sustained disagreements on outputs. Although there were several differences of opinion expressed at the beginning of the process, the experience was that, after discussion, opinions were often closer than originally thought and agreement could be reached. The research team reviewed and considered the input from the ERG in the light also of the current developmental and clinical literature, just as the ERG scrutinised recommendations for relevance and acceptability.

### Stages of co‐design and adaptation

#### Phase 0: Study set up and literature review

During this phase, the ERG was formed, given training, and had orientation meetings (Figure 1). Research ethics application for the embedded qualitative study was created. The research team reviewed and synthesised the academic literature on autistic anxiety problems (Vasa et al., 2020), evidence on existing effective interventions for autistic child anxiety (Sharma et al., 2021; Spain & Happé, 2020), and NICE guidance (GDG178, 2013). For independent review of the available literature, the local NHS library services conducted a separate search. See supporting Information (SI pages 2–5) for a more detailed description of the literature shared.

***Expert reference group Training sessions*** Separate training sessions were held (chaired by the ERG chairs) for the clinicians and the non‐clinical ERG members (those with lived experience). As this was prior to the establishment of the ERG, members of the research team, including PPI and autistic members, decided together to hold the training sessions separately. This was done to promote open discussion of the power dynamics, perceived challenges, fears and worries about working together, and explore biases in such self‐contained groups. Members of the research team joined for part of both training sessions to introduce themselves and answer questions about the research process. The training sessions outlined the role of the ERG, the project timeline, and the role of the research team. Expert reference group members were provided with a welcome pack in the post following on from the training, which included primary source material on OSI written by CC.

***Expert reference group meetings 1&2*** During these meetings, focus was placed on building the group, co‐creating group rules, understanding the anxiety intervention, establishing ways of working and reviewing ethics documentation.

#### Phase 1: Evidence gathering

***Expert reference group activity*** In meetings three and four, the ERG critically evaluated the summary of recommended therapeutic adaptations for autistic children. They recommended a more diverse literature for exploration, including from critical autism studies (Heselton, 2021; Milton, 2018), and the neurodiversity movement (Dyck & Russell, 2020; Leadbitter et al., 2021). Expert reference group contributed their thoughts on what was needed to support autistic children with anxiety problems and their families and their responses to recommended adaptations are summarised in Table S2.

***The Embedded Qualitative*** ***study*** as above was conducted during phase 1. Emerging themes from the qualitative analysis were added to the key insights documents, reviewed by the research team and within ERG meetings five and six, as below (Figure 1, and see SI page 4).

#### Phase 2: Content writing, consolidation and focus group consultation

Phase 2 focussed on writing the key content and solidifying the required adaptations, as well as checking the acceptability of the intervention with multiple stakeholders (see Figure 1). The ERG was presented with data from the qualitative analysis during meetings five and six, which was then fed back to the Research Team, and into the intervention adaptation. During meetings seven, eight and nine, the ERG reviewed a blueprint of the adapted intervention. Following meeting seven, the intervention plan was taken to two focus groups of parents of autistic children with anxiety problems, to gain further opinions and insights into the acceptability of the intervention. During meetings eight and nine the findings from these focus groups were shared with the ERG and then research team and the blueprint was further refined. During meeting ten a final outline of the adapted intervention was established; next steps were discussed and outcome measures for future trials reviewed. During this time the research team was also working on the funding application for further research time to digitise and test the intervention.

## RESULTS

### Recommendations from the literature

The academic literature makes several recommendations for key strategies that can be used to adapt CBT interventions to meet the needs of autistic people (Spain & Happé, 2020), and NICE Autism Guidance (National Institute for Health and Care Excellence, 2013) itself includes specific recommendations for such adaptation (see SI pages 2–5). The academic recommendations, well summarised by the NICE (2013) guideline, have mostly been integrated into the adapted intervention. The lived experience perspective provided additional information on how to present information, which components felt the most important, and additional family perspectives.

### Key insights from the co‐design process (see also Table S3)

***Neurodiversity based Autism information*** The ERG felt strongly that well‐integrated neuro‐affirmative information was needed throughout the intervention. To be acceptable, programme ethos needed to clearly centred around not trying to change autistic children, but to give parents the tools to support their children to manage their anxiety problems. Consensus of the ERG and research team, informed by the Qualitative Study, was that psychoeducation needs to focus on how common autistic needs interface with anxiety problems, including information on sensory needs and how they relate and do not relate to anxiety problems, interoception differences, autistic shutdown and overwhelm, the up‐and‐down nature of anxiety for autistic children, and understanding daily underlying stressors.

***Personalisation*** The ERG highlighted the importance of thinking about the individual child and the need for personalisation throughout the programme rather than a ‘one size fits all’ approach. Expert reference group members reflected together, informed by the Qualitative Study, on how hard it can be for the parent to understand the underlying elements of their child's difficulties and how best to support their child. The ERG identified that without bringing the content back to the individual child and their needs it would be easy to misinterpret elements of the child's needs and resources. This is consistent with the ethos of the original OSI programme and part of the rationale for maintaining the format of an individual self‐directed programme (with embedded activities that support personalisation) with therapist support.

***The importance of being understood*** The ERG, informed by the Qualitative Study, emphasised the importance of therapists understanding how challenging anxiety can be and acknowledging that the whole family, including siblings, are involved and impacted by the anxiety problem. The conversation with the ERG highlighted that importance should be placed on helping parents support their child, whilst understanding and validating the challenges and pressures parents are managing.

***Pressure to conform to neurotypical expectations*** ERG members, considering also the Qualitative Study results, reflected perceived and explicit pressures from school, internal expectations, and the wider family for the child to conform to non‐autistic expectations. This was identified by the research team as particularly relevant when helping parents set goals and helping parents think about their child's independence, specifically helping parents to think about what they feel their child “should” do, what their child needs to do, what will be helpful for them to do. Support to set realistic goals and giving parents a protected place to think about their child's needs was highlighted as important, as was a place to think about talking and working with schools.

***Cognitive Behavioural Therapy*** The ERG expressed initial concern around CBT approaches. During the co‐design process, it became clearer that the concern was around CBT application by therapists without adequate knowledge of autism. The ERG, reflecting also some Qualitative Study results, were also concerned that some strategies initially framed for non‐autistic children could potentially reinforce narratives that autistic thinking was ‘wrong’ and that children should try to think and feel more like their non‐autistic peers. They highlighted the importance of integrating a neurodiversity affirmative approach alongside classic CBT techniques.

## DISCUSSION

The experience of co‐design in this study demonstrated how multiple perspectives could be integrated into a consensus on an adapted mental health intervention. Anxiety presentation is a complex phenomenon, and the evolution of anxiety conditions in autistic development is likely to be different in important ways from that in non‐autistic development. Longitudinal study (Shephard, et al., 2019) shows autistic and anxiety phenomena developing simultaneously in early development, suggesting that some features of autistic development may be inherently anxiogenic, for instance intrinsic biologically in terms of the effects of neurodiversity on sensory experience, or on impacts of early interpersonal experience (Stevenson‐Hinde, et al., 2013). This may then be compounded as the child grows by the stresses of difference in relation to many aspects of neurotypical social environments. All these features need considering within intervention development. The co‐design process, including autistic young people, autistic adults, parents, clinicians and researchers brought to bear a variety of experience and expertise to make an adaptation that combines the current evidence and views and values of members of the autism community. This paper, following reporting protocol for PPI in research (Table S4), illustrates the process of the co‐design and positive and negative features associated with it.

What worked well (identified by the ERG and research team):

1. Specifying ERG co‐chairs across clinical and user experience was important to the functioning of the group; whose composition was also valuably diverse and covered major stakeholder groups with varied experience.
2. The initial ERG training and on‐going support procedures were valuable and necessary to consolidate the group and their confidence in their role.
3. Independence between ERG and Research Team during the work. Beyond the initial introductions and discussion, all the interaction was mediated through the co‐chairs, along with one autistic parent (DM) common to both groups. This process minimised undue influence that researchers might have had on the group working.
4. Detailed reciprocal iterative dialog between ERG and Research Team from the beginning of the project (Figure 1, Table S1), which ensured that co‐design was initiated at the outset and sustained productively throughout. The aimed‐for effect was to allow diverse viewpoints to be expressed with confidence and for equitable moderation of any disagreements. Clinicians, researchers and experts by experience will inevitably bring their own perspectives to the challenge, and we were encouraged in the project that the dialogic nature of this process allowed these to be confidently expressed and also often resolved as discussion around details proceeded, with pre‐conceptions on all sides often modified in the process.
5. Appropriate payment for the ERG members for all elements of contribution
6. Consideration of different styles and communication needs across meetings and out of meeting communications, including flexibility on length of attendance if fatigued.
7. On‐going reflective meetings between ERG co‐chairs to ensure ERG input was at the forefront of all decisions. These provided a place to regularly review power differentials and what was working well and what wasn't in the ERG meetings. These reflection meetings were key to the facilitation style employed by the ERG chairs, which aimed for a flexible, non‐critical, non‐defensive stance throughout.

These features seemed overall an efficient and respectful procedure for co‐design; the ERG and research team both reflected that debates around constructs and prior assumptions allowed for the open exploration of what was needed in the intervention, avoiding unproductive conflicts. The team was unequivocally encouraged in the end by the added value accrued from this process, including a shift of what to focus on in the intervention content, the use of neurodiversity affirmative language and understanding of the challenges and pressures for parents.

***Challenges*, *Limitations and Learning*** The study, particularly in its start‐up phase, was inevitably more time‐consuming than if experts by experience had not been integrated into the design (Oliver et al., 2019). Similarly, the iterative nature of the project as well as integrating multiple perspectives took longer than a traditional academic literature review. Both ERG and research team found that the additional time needed was more than compensated by the value generated from the co‐design and could be mitigated by careful planning and allowing for longer lead times to facilitate engagement.

There was a limitation in terms of ERG diversity. Although a neurodiverse and experientially diverse group, the ERG was predominantly white British and there were limitations in ethnic, socioeconomic, sexuality/gender and cultural diversity. This represents a more general issue; people from communities that are an ethnic minority in the UK are under‐represented in presentations to autism clinical services (Russell et al., 2011), in autism research, and in many aspects of autism charity and advocacy work; they were not represented in expressions of interest for ERG membership. This could have been potentially mitigated by a lengthier ERG recruitment period if funding had allowed. More representation of intersectional experience would have enhanced this project and more work generally is needed in the autism field to promote more diverse service access, participation, and co‐production.

### Future research

In the light of the lag‐time between concept and implementation of evidenced intervention practice, more co‐design work of this kind is urgently needed to inform inclusive, sophisticated, and neuro‐affirmative interventions for the future. There may inevitably still be hesitation in clinical research teams about embarking on unfamiliar co‐design processes, including whether the benefits will outweigh the additional time and cost. Also, whilst there is a strong argument in principle for doing this, it is also still to be demonstrated whether interventions developed in this way will prove empirically more acceptable and effective in clinical practice. More research attention is therefore needed to extend this type of work and test the effectiveness of co‐designed interventions. This will further develop clinical intervention practice, as well as providing more understanding of co‐design methods and their benefits or drawbacks. To support this, research commissioners will need to prioritise and accept the funding implications of co‐design and participatory methods.

## AUTHOR CONTRIBUTIONS

**Tasha Cullingham**: Conceptualization; data curation; funding acquisition; investigation; methodology; project administration; supervision; writing – original draft; writing – review & editing. **Una Rennard**: Conceptualization; data curation; funding acquisition; writing – review & editing. **Cathy Creswell**: Conceptualization; funding acquisition; methodology; supervision; writing – review & editing. **Michael Larkin**: Conceptualization; formal analysis; funding acquisition; writing – review & editing. **Damian Milton**: Conceptualization; data curation; funding acquisition; writing – review & editing. **Lucie Godber**: Conceptualization; writing – review & editing. **Karen Leneh Buckle**: Data curation; methodology; writing – review & editing. **Kate Gordon**: Data curation; writing – review & editing. **Jonathan Green**: Conceptualization; funding acquisition; methodology; supervision; writing – review & editing.

## CONFLICT OF INTEREST STATEMENT

Cathy Creswell is the author of a book for parents used by clinical teams to deliver parent‐led CBT and she receives royalties from sales. Cathy Creswell is a developer of the OSI platform for digitally augmented parent‐led CBT but she does not receive any personal financial benefits from the use of OSI. No other authors declare conflict of interests.

## ETHICAL CONSIDERATIONS

This study received ethical approval from the NHS Health Research Authority (IRAS project ID 307932, REC Reference 22/HRA/1600, 25th April 2022). The sponsor was Manchester University NHS Foundation Trust and the study was undertaken in accordance with standards in the Declaration of Helsinki.

## Supporting information

Supporting Information S1

## Data Availability

Research data are not shared for reasons of confidentiality.
