# Supplementary material for: The process of co‐design for a new anxiety intervention for autistic children
Source: JCPP Adv. 2024 Aug 13;5(2):e12255. doi: 10.1002/jcv2.12255 (PMC12159306; doi:10.1002/jcv2.12255)
Supplement: Supplementary file 1 — Supporting Information S1 [file JCV2-5-e12255-s001.docx]

**THE PROCESS OF CO-DESIGN FOR A NEW ANXIETY INTERVENTION FOR AUTISTIC CHILDREN**

Cullingham T, Rennard U, Creswell C, Milton DM, Buckle KL, Godber L, Gordon K, Larkin M, Green J.

**SUPPORTING INFORMATION**

1. **Methods in the embedded Qualitative Study**

***Sampling and Participants***The study used convenience/purposive sampling, recruited via social media, charities, parent-carer forums and schools. Participants were selected from an open call via local and national networks, explaining the study and asking for applications if they would like to be interviewed or participate in a focus group. 10 parents of autistic children with anxiety problems (children age 8-12 years, median age 10) and 9 autistic children with anxiety (children age 8-12 years, median age 10) problems were interviewed in this study.  Four parents of autistic children with anxiety participated in the focus groups. All participants lived in the UK and all completed screening, two child participants were deemed ineligible due to not currently experiencing clinically significant levels of anxiety.

***Interviews*** Semi-structured interviews were conducted between May 2022-January 2023. Participants were given the choice between virtual or in-person interviews. Prior to analysis, all transcripts were anonymised, and pseudonyms used.

The parent interviews focused on the experience of parenting an autistic child with anxiety problems, how they respond to their child’s anxiety, and their experiences of accessing support for their child.

The child interviews focused on the child’s experiences of anxiety, what helps when they are anxious, what it is like to talk about anxiety, and how their parents respond to their anxiety. All children were given the choice as to whether a parent would be present during the interview.

**Child Interview Outline Schedule:**

1. Do you know what I mean when I say anxiety or worries?
2. Can you tell me what worries feel like?
3. Are some worries worse than others?
4. When do you feel the most worried?
5. How do you know when you are worried?
6. What types of things worry you?
7. How often do you feel worried?
8. What makes you feel better when you are worried?
9. Do you talk to your parents about worries?
10. What do your parents do that helps your worries?
11. What do you parents do that make your worries worse
12. Do you think your parents know when you are worried or scared?
13. What do you want people to know about your worries?
14. What helps when you are worried?
15. What is it like to have autism and worries?

**Parent Interview Outline Schedule:**

1. Please describe your child.
2. Please tell us about your child’s challenges.
3. Please tell us about your child’s anxiety.
4. How do you know your child struggles with anxiety?
5. What does your child look like when they are anxious?
6. What do you think causes your child’s anxiety?
7. Do you think your child understands their anxiety?
8. What types of situations make your child anxious?
9. How do you respond when your child is anxious?
10. What helps when your child is anxious in the moment?
11. What do you think helps your child overcome their anxiety longer term?
12. What do you think is important for parents of autistic children with anxiety to know?
13. What is your understanding of anxiety management?
14. What types of support have you been offered?
15. What types of support would be helpful to you?
16. What would be most important for a professional supporting you to support your child with anxiety to know?
17. Is there anything that would stop you from accessing support to help you child manage their anxiety?

***Analysis*** Qualitative analysis consisted of Interpretative Phenomenological Analysis (IPA) and Template Analysis (TA) for both the parent and the child interviews. Case-level analysis using IPA was used for five child and parent interviews. Two researchers worked together to create person level, and then group level themes for each of the transcripts undergoing IPA. These emergent themes were considered by the ERG in their Meetings 5/6 (see Figure 1) and finalised following consensus discussion. A template was developed from the group level themes which was then extended to the remainder of the parent and child samples using TA, adapting the template as new insights arose.

Themes from the qualitative analysis were then used alongside further input form the literature and subsequent ERG meetings in order to underpin the adaptation to the interventions. Focus groups: the focus group data was not analysed via IPA or template analysis, but instead used as a checkpoint for the validity of the content and to further have the content reviewed by the target population. All data was recorded and transcribed and key themes and findings recorded.

**Exemplar material from Child Interviews**

Anxiety is annoying and hard to make sense of.

Things get rid of my anxiety in the short term, but it comes back.

I am constantly fending off impending doom.

I doubt my own experience, am I right about what I feel?

I feel unsafe when things are unpredictable.

In order to survive I need to hide who I am, and what I feel.

My family is important in helping me with my anxiety.

**Examplar material from Parent Interviews**

It is very hard. Helping my child get through the day takes tremendous effort.

My child’s anxiety is extreme and horrific to see.

I am on a constant “journey of learning” to understand my child and their needs.

To be helpful you need to understand

I am my child’s safe person.

Feelings are connected; emotional regulation starts with me.

I need to help my child so they can cope with things when they are older.

My child does things differently than society and I expected.

We have been let down by services.

1. **Literature Review on intervention amendments in discussed with ERG**

Some characteristics of Autism (such as difficulties with social communication/emotional recognition/abstract thinking) mean autistic people may require adaptations to standard evidence-based psychological treatments to adequately meet their needs (NICE, 2012). Evidence suggests that adapted psychological interventions, particularly CBT, may be more effective for autistic people, compared to the standard forms (Wood et al., 2020). CBT designed for non-autistic individuals does not consider the specific needs of autistic individuals, nor consider autistic strengths that may be important for intervention success.

***Further suggestions from the literature for Autism adaptions:***

- Providing written/visual outlines of the session agendas (Spain and Happe, 2020)
- Offering positive feedback (Spain and Happe, 2020)
- Tailor session to unique needs of the individual (Spain and Happe, 2020)
- Use terminology appropriate to the individual’s level of comprehension (Spain and Happe, 2020)
- Use of formulation to guide intervention choice (Spain and Happe, 2020)
- Children who are more sensitive to reward contingencies may benefit more from approaches that work more explicitly with reward (Hollocks et al., 2022)
- Avoiding metaphors (Cooper et al. 2018)
- Use of coping skills to manage feelings of anxiety (Hallett et al., 2021)
- Use of a ‘toolbox’ for emotional regulations for example relaxation, special interests, social, and physical tools for children to use in different situations (Oerbeck et al., 2018)
- **The PRECISE Model** (Donoghue, Stallard and Kucia., 2010)

**Adaptions within parent-mediated adapted CBT interventions** Hallett et al. (2021)

- Parents are encouraged to model anxiety coping skills, to reduce reassurance, and to increase emotional labelling.
- Intolerance of uncertainty encouraged by parents, for example parents are encouraged to introduce periods of change during the day to increase child’s flexibility.
- Encouraging parent wellbeing and self-care.
- Aspects of ‘mindful parenting’, encouraging parents to ‘step back’ and respond to

their child with awareness (in a predictable way), even in stressful situations.

**5 key areas of adaption (**Moore and Davies, (2010))

1. Disorder specific hierarchies: Important not to focus only on the presenting problem (i.e., anxiety) but also disorder specific difficulties such as communication and social skills training.
2. Concrete and visual tactics: Adjusting materials to be more developmentally appropriate. Inclusion of are emotion statements, pictures and drawings, visual worksheets, narratives/social stories, and role play.
3. Child specific interests: to facilitate motivations to engage
4. Parent involvement: reinforces positive behaviour via practice and reward at home. Also, useful to encourage parent wellbeing.
5. Environment: Adapt length of session, sensory requirements, and reasonable adjustments

***Key studies and resources:***

- Cooper, K., Loades, M. E., & Russell, A. (2018). Adapting psychological therapies for autism. Research in autism spectrum disorders, 45, 43-50.
- Donoghue, K., Stallard, P. and Kucia, J. (2010) The Clinical Practice of Cognitive Behavioural Therapy for Children and Young People with a Diagnosis of Asperger’s Syndrome, Clinical Child Psychology and Psychiatry, Vol. 16 (1), pp. 89-102.
- Hallett, V., Mueller, J., Breese, L., Hollett, M., Beresford, B., Irvine, A., ... & Simonoff, E. (2021). Introducing ‘Predictive Parenting’: A feasibility study of a new group parenting intervention targeting emotional and behavioral difficulties in children with autism spectrum disorder. Journal of Autism and Developmental Disorders, 51(1), 323-333.
- Hollocks, M. J., Wood, J. J., Storch, E. A., Cho, A. C., Kerns, C. M., & Kendall, P. C. (2022). Reward Sensitivity Predicts the Response to Cognitive Behavioral Therapy for Children with Autism and Anxiety. Journal of Clinical Child & Adolescent Psychology, 1-8.
- Oerbeck, B. , Overgaard, K. R. , Stein, M. B. , Pripp, A. H. , & Kristensen, H. (2018). Treatment of selective mutism: A 5‐year follow‐up study. *European Child and Adolescent Psychiatry*, 27(8), 997–1009. 10.1007/s00787-018-1110-7
- Moree, B. N., & Davis III, T. E. (2010). Cognitive-behavioral therapy for anxiety in children diagnosed with autism spectrum disorders: Modification trends. Research in Autism Spectrum Disorders, 4(3), 346-354.
- National Institute for Health and Care Excellence. (2012). Autism spectrum disorder in adults: Diagnosis and management. NICE guideline [CG142].
- Sofronoff, K., Attwood, T., & Hinton, S. (2005). A randomised controlled trial of a CBT intervention for anxiety in children with Asperger syndrome. Journal of child psychology and psychiatry, 46(11), 1152-1160.
- Spain, D, and Happé F 2020. “How to Optimise Cognitive Behaviour Therapy (CBT) for People with Autism Spectrum Disorders (ASD): A Delphi Study.” Journal of Rational-Emotive and Cognitive-Behavior Therapy: RET 38 (2): 184–208.
- Storch, E. A., Wood, J. J., Guzick, A. G., Small, B. J., Kerns, C. M., Ordaz, D. L., ... & Kendall, P. C. (2022). Moderators of response to personalized and standard care cognitive-behavioral therapy for youth with autism spectrum disorder and comorbid anxiety. Journal of Autism and Developmental Disorders, 52(2), 950-958.
- Walters, S., Loades, M., & Russell, A. (2016). A systematic review of effective modifications to cognitive behavioural therapy for young people with autism spectrum disorders. Review Journal of Autism and Developmental Disorders, 3(2), 137-153.
- Wood JJ, Kendall PC, Wood KS, et al. Cognitive Behavioral Treatments for Anxiety in Children With Autism Spectrum Disorder: A Randomized Clinical Trial. JAMA Psychiatry. 2020;77(5):474–483. doi:10.1001/jamapsychiatry.2019.4160.

| **Supplementary Table 1: AASPIRE Guidance on co-production with autistic people and community groups** | | |  |
| --- | --- | --- | --- |
| **The Academic Autism Spectrum Partnership in Research and Education (AASPIRE) guidance on involving autistic adults as co-researchers** | | |  |
| **Guidance** | **Implemented** | **Examples** |  |
| Be transparent about partnership goals and choose an appropriate approach to match those goals | Yes | 1. Research plan was co-created with the research team, including PPI members. Considering how best to engage community members and consider different levels of engagement and involvement.  2. Project goals were shared from the beginning with the ERG. Clearly outlining the commitment, responsibility and ways of working, as well as limitations around participation and challenges. |  |
| Clearly define community partner roles, consider who needs to be included on the team and partner with people who are likely to help the project succeed | Yes | 1. The expertise within the group was carefully considered with the PPI representatives. We invited, autistic leaders of community groups, academics, parents of autistic people and young autistic people with anxiety. |  |
| Create processes for effective communication and power sharing | Yes | 1. Meeting structure was co-created within the ERG.  2. Meetings were co-chaired between PPI lead and lead researcher.  3. Breaks were given and the structure of breaks based on ERG participants needs.  4. Meeting structures was flexible and the meeting plan shifted from long, infrequent meetings to shorter and more frequent meetings.  5. PPI lead led discussions around critiquing the literature.  6. All participants expertise was valued and all there was no held “correct” position or beliefs.  7. all ERG members were sent welcome backs with a copy of the Helping your child change book, a branded study notebook, information about the study and the research team members. |  |
| Regularly focus on building and maintaining trust | Yes | 1. Open and honest (including leads) team reflections occurred at the beginning and the end of every meeting.  2. Open sharing of challenges and limitations, by research team with ERG members.  3. Listening and checking that points made by ERG had been heard correctly understood by research team.  4. ERG Minutes checked and shared with all ERG members, before being shared with research team. As this is the ERG’s information the ERG had final say and could request changes and request for information to be removed. |  |
| Collaboratively disseminate findings | Yes | 1. All ERG members invited to co-author on this and other papers.  2. Co-creation of lay summaries.  3. Co-writing of all outgoing communications including recruitment documents. |  |
| Actively encourage community capacitation | Yes | 1. Opportunities were provided for autistic individuals as well as parents of autistic individuals to engage as researchers.  2. Supporting leading members of the autistic community and younger members to input equally.  3. Engaging with several other autistic community groups not embedded within this study in the form of additional outreach sessions. |  |
| **Reflections from the Authentistic Research Collective** | | |  |
| **Guidance** | **Implemented** | **Examples** |  |
| Group rules and traffic light system | Yes | 1. Collaborative group rules were established at the first meeting  2. We did not implement an official traffic light system. We instead used a check-in system at the beginning of the meetings, which was co-constructed with the ERG. We also had an opt in system for involvement, so that ERG members could provide as much or as little participation and input as they felt up to. |  |
| Adapting the environment to suit people’s needs | Yes | 1. Use of on-line meeting structures for people to access and manage their own environmental needs.  2. Use of chat-box, camera on or off, and regular breaks throughout the meetings.  3. PowerPoints were used to summarise content as well as written material provided beforehand.  4. breaks provided as needed as well as regularly built into meetings. |  |
| Inclusion of digital communication tools | Yes | 1. All meetings were held online  2. No communication was given priority, chat, verbal speaking, and use of reactions on zoom, were given equal space. Time to wait and think was provided so people could add into the chat box without being distracted or spoke over. |  |
| Encouraging group members to feel they are authentic *(creating a safe space to be authentic)* | Yes | 1. Open and honest approach was taken by all. Research and professional members were expected to share, be vulnerable and honest to create a more authentic experience.  2. All members of the research team attended for a portion of either training or initial session to introduce themselves. The research team were provided to all ERG members, including information on what they enjoyed outside of work. |  |
| Supporting autistic strengths | Yes | 1. The ERG together rejected the diagnostic conceptualisation of Autism viewed only through a deficit focused lens and moved towards a social model of disability.  2. All members’ expertise, experience and understanding whether as an autistic individual, a parent of an autistic individual, research or clinician was valued and considered to be an experience worth understanding. The group also shared an awareness that all experience was valuable but also limited to our individual perspectives. |  |

**Supplementary Table 2 - Elements from NICE guidance for amending interventions and their use in co-design**

| **Elements within NICE Guidelines** | **Incorporated in the adapted intervention** | **Rationale** |
| --- | --- | --- |
| Emotion recognition training | No | Conversations with the ERG highlighted that they would not be comfortable with this being included. The conversations centred around how this could reinforce the non-autistic lens of how emotions should feel, and that autistic people should try and assimilate to present as non-autistic.  Qualitative data [collected](https://paperpile.com/c/MCvh5B/2bxn) within this project suggested children did not feel as though they were the authority on their internal experiences. Thus, training a non-autistic view on emotion regulation may reinforce a feeling of wrongness.  The research team identified this would add an additional demand on parents to support them to navigate these discussions and do this work with their child.  Although included in many CBT interventions for anxiety problems, there is limited evidence that this is an integral mechanism of change.  This was not the core purpose of the intervention. |
| Greater use of written and visual information and structured worksheets | Yes | The ERG and research team felt that the inclusion of this component would be important. They felt that providing different ways to talk about anxiety, including visual depictions, would be helpful.  The importance of choice was highlighted by the ERG. Discussions highlighted empowering parents to think about what might work best with their child. |
| A more cognitively concrete and structured approach | Partially | This contributed to OSI being selected for adaptation as the original version contains a step-by-step structured plan. Additionally, there will be structured worksheets for parents. Parents will be asked to think about what works for the parent and child dyad. |
| Simplified cognitive activities, for example, multiple choice worksheets | No | Not relevant as we are not working directly with the child and will not be directly engaging with cognitive activities with the child.  We will provide parents with structured and simplistic worksheets, as well as multimodal content to increase accessibility so that the intervention will be accessible to autistic and non-autistic parents. |
| Involving a parent or carer to support the implementation of the intervention, for example, involving them in therapy sessions | Yes | This contributed to OSI being selected for adaptation as it is a parent-led intervention. |
| Maintaining attention by offering regular breaks | No | Not relevant as not working directly with the child.  We will include information on when to talk to children about anxiety problems and parental consideration of their child’s preferred communication and attentional needs will be considered.  We will encourage all therapists to think about accessibility needs to support parents to access the programme. |
| Incorporating the child or young person’s ‘special interests’ into therapy if possible | Yes | Whilst working with parents we will be thinking about how to include the child’s interests to support them to engage. Drawing on parents’ expertise on how best to engage the young person.  Discussions with the ERG centred around the use of the word ‘special’ as being derogatory and dismissive and the terminology was viewed as pathologizing an interest. Therefore, the term ‘special’ has been removed and we simply speak about a child’s interests. |

**Supplementary Table 3 - Key Intervention Adaptations following co-design**

| **Key adaptations** | | |
| --- | --- | --- |
| **Key Adaptation** | **Summary** | **Source** |
| **Inclusion of Neuro-diversity affirmative language and identify first language** | The adapted intervention uses identify first language and this has been integrated throughout. Additionally autistic characteristics such as communication, social and sensory differences have been described and identified as differences and not necessity deficits, throughout. | ERG  Literature on the neurodiversity movement  Critical autism studies |
| **Autism specific psychoeducation** | Including how anxiety presents and is experienced by autistic people | ERG  Qualitative study  Literature on adapting CBT to support autistic people  Critical autism studies  Neurodiversity literature |
| **Additional communication strategies** | Providing parents with concrete communication strategies and information about talking about feelings | NICE guidelines |
| **Information about environmental goodness of fit** | Including information on sensory needs and environmental goodness of fit | ERG  Critical Autism studies  Neurodiversity literature  Clinical experience |
| **Acknowledging the challenges of supporting a neurodiverse child in a neurotypical world** | Acknowledgement of the challenges parents face and normalising this. | ERG  Qualitative Study |
| **Providing more and an additional check in session** | Providing slightly more time in the intervention to problem solve and provide parents with an additional check in call. | ERG  Qualitative study  NICE guidelines  Literature on CBT to support autistic individuals. |
| **Information on the unique developmental trajectories of autistic children** | Helping parents think about their child’s potential uneven profile of needs and skills. Thinking about how this may be different for autistic children than non-autistic children. | ERG  Qualitative study  Developmental literature on Autistic children |

**Supplementary Table 4 – GRIPP2 long form checklist for involvement of patients and public**

Staniszewska S, Brett J, Simera I, Seers K, Mockford C, Goodlad S et al. GRIPP2 reporting checklists: tools to improve reporting of patient and public

involvement in research *BMJ*2017; 358 :j3453 doi:10.1136/bmj.j3453

| **Section and topic** | **Item** | **Reported on page No** |
| --- | --- | --- |
| Section 1: Abstract of paper | |  |
| 1a: Aim | Report the aim of the study | 2 |
| 1b: Methods | Describe the methods used by which patients and the public were involved | 2 |
| 1c: Results | Report the impacts and outcomes of PPI in the study | 2 |
| 1d: Conclusions | Summarise the main conclusions of the study | 2 |
| 1e: Keywords | Include PPI, “patient and public involvement,” or alternative terms as keywords | 2 |
| Section 2: Background to paper | |  |
| 2a: Definition | Report the definition of PPI used in the study and how it links to comparable studies | 3 |
| 2b: Theoretical underpinnings | Report the theoretical rationale and any theoretical influences relating to PPI in the study | 3,4,5 |
| 2c: Concepts and theory development | Report any conceptual models or influences used in the study | 3,4,5 |
| Section 3: Aims of paper | |  |
| 3: Aim | Report the aim of the study | 2,3,4,5 |
| Section 4: Methods of paper | |  |
| 4a: Design | Provide a clear description of methods by which patients and the public were involved | 5 |
| 4b: People involved | Provide a description of patients, carers, and the public involved with the PPI activity in the study | 6,7 |
| 4c: Stages of involvement | Report on how PPI is used at different stages of the study | 8,9,10 |
| 4d: Level or nature of involvement | Report the level or nature of PPI used at various stages of the study | 8,9,10 |
| Section 5: Capture or measurement of PPI impact | |  |
| 5a: Qualitative evidence of impact | If applicable, report the methods used to qualitatively explore the impact of PPI in the study | 11,12, SM Tables 1,2,3 |
| 5b: Quantitative evidence of impact | If applicable, report the methods used to quantitatively measure or assess the impact of PPI | na |
| 5c: Robustness of measure | If applicable, report the rigour of the method used to capture or measure the impact of PPI | na |
| Section 6: Economic assessment | |  |
| 6: Economic assessment | If applicable, report the method used for an economic assessment of PPI | na |
| Section 7: Study results | |  |
| 7a: Outcomes of PPI | Report the results of PPI in the study, including both positive and negative outcomes | 11,12 SM Table 1,2,3 |
| 7b: Impacts of PPI | Report the positive and negative impacts that PPI has had on the research, the individuals involved (including patients and researchers), and wider impacts | 11,12 |
| 7c: Context of PPI | Report the influence of any contextual factors that enabled or hindered the process or impact of PPI | 11,12 |
| 7d: Process of PPI | Report the influence of any process factors, that enabled or hindered the impact of PPI | 11,12, Fig 1 |
| 7ei: Theory development | Report any conceptual or theoretical development in PPI that have emerged | 11,12 |
| 7eii: Theory development | Report evaluation of theoretical models, if any | na |
| 7f: Measurement | If applicable, report all aspects of instrument development and testing (eg, validity, reliability, feasibility, acceptability, responsiveness, interpretability, appropriateness, precision) | na |
| 7g: Economic assessment | Report any information on the costs or benefit of PPI | na |
| Section 8: Discussion and conclusions | |  |
| 8a: Outcomes | Comment on how PPI influenced the study overall. Describe positive and negative effects | 13,14,15 |
| 8b: Impacts | Comment on the different impacts of PPI identified in this study and how they contribute to new knowledge | 13,14,15 |
| 8c: Definition | Comment on the definition of PPI used (reported in the Background section) and whether or not you would suggest any changes | 15 |
| 8d: Theoretical underpinnings | Comment on any way your study adds to the theoretical development of PPI | 15 |
| 8e: Context | Comment on how context factors influenced PPI in the study | 15 |
| 8f: Process | Comment on how process factors influenced PPI in the study | 13,14 |
| 8g: Measurement and capture of PPI impact | If applicable, comment on how well PPI impact was evaluated or measured in the study | na |
| 8h: Economic assessment | If applicable, discuss any aspects of the economic cost or benefit of PPI, particularly any suggestions for future economic modelling. | na |
| 8i: Reflections/critical perspective | Comment critically on the study, reflecting on the things that went well and those that did not, so that others can learn from this study | 13,14,15 |
